# Woman experiencing gynecologic surgery: coping with the changes imposed by surgery**^1^**

**DOI:** 10.1590/1518-8345.1081.2780

**Published:** 2016-08-29

**Authors:** Carolina de Mendonça Coutinho e Silva, Octavio Muniz da Costa Vargens

**Affiliations:** 2 Doutoranda, Faculdade de Enfermagem, Universidade do Estado do Rio de Janeiro, Rio de Janeiro, RJ, Brazil. Enfermeiro, Hospital do Câncer III, Instituto Nacional de Câncer, Rio de Janeiro, RJ, Brasil.; 3 PhD, Professor Titular, Faculdade de Enfermagem, Universidade do Estado do Rio de Janeiro, Rio de Janeiro, RJ, Brazil.

**Keywords:** Nursing, Women's Health, Gynecologic Surgical Procedures, Femininity

## Abstract

**Objectives:**

to describe the feelings and perceptions resulting from gynecologic surgery by women and analyze how they experience the changes caused by the surgery.

**Method:**

a qualitative, descriptive and exploratory study, which had Symbolic Interactionism and Grounded Theory as its theoretical framework. Participants of the study: 13 women submitted to surgery: Total Abdominal Hysterectomy, Total Abdominal Hysterectomy with bilateral Adnexectomy, Wertheim-Meigs surgery, Oophorectomy, Salpingectomy, Mastectomy, Quadrantectomy and Tracheloplasty. Individual interviews were conducted, recorded and analyzed according to the comparative analysis technique of the Grounded Theory.

**Results:**

from the data two categories emerged - Perceiving a different body and feeling as a different person and; building the meaning of mutilation. The changes experienced make women build new meanings and change the perception of themselves and their social environment. From the interaction with their inner self, occurred a reflection on relationships, the difference in their body and themselves, the functions it performs and the harm caused by the surgery.

**Conclusions:**

the participants felt like different women; the mutilation developed in concrete feelings, due the loss of the organ, and in abstract, linked to the impact of social identity and female functionality. The importance of the nurse establishing a multidimensional care, to identify the needs that go beyond the biological body is perceived.

## Introduction

The diseases of the genitourinary apparatus and breast are responsible for a large number of surgical procedures throughout the world. Hysterectomy, for example, is a gynecologic surgery performed in most developed countries^1^, and as well as mastectomy is widely used as treatment for women, because breast cancer is the main disease among women in Brazil and in the world^2^. The diseases of the genitourinary apparatus and mammary glands not only are a serious public health problem, but also affect organs that have symbolic values and are related to femininity^3^ ^-^ ^4^. 

For every woman, gynecological surgery is an experience lived in a particular way. The sensations and perceptions that occur with the woman after the removal of an organ from the reproductive system are identified in this study, showing that women face a number of changes in their body and in their self (self-consciousness). 

It is known that there are feelings and characteristics related to the perception of the body that are common to women after surgery: strangeness and modification of the body image (3-4), perception of an altered and different body ^5^ ^-^ ^6^, feeling of mutilation of her body^5^, feeling of emptiness, of being different from other women and the perception of a marked body^3^ ^,^ ^7^. It means that physically, this woman cannot perceive herself as the same anymore.

Each person is situated in the world through the body and therefore it would be impossible to separate the feelings of the body and vice versa. In the body we record and carve the marks of our existence, resulting in a form and manner that people are inserted in the world^8^. What happens to the body is reflected within the individual, as well as face features, countenances and body attitudes change with feelings and emotions.

The body represents to the world what women are, so the loss of a body part has a big impact on their way of communicating with the outside world and with themselves. Some women have a positive attitude to face the situation, accepting the new body image and establishing a new form of communication with the world. Others, however, experience the loss as a triggering factor of insecurity in their relationships, as a new self-image is formed and this is linked to a bad experience^6^ ^-^ ^7^ ^,^ ^9^ ^-^ ^11^.

Given the large number of women who annually undergo gynecological surgery due to diseases in the genitourinary apparatus and breast, it is necessary to consider the various issues that are involved in the care of these women.

When planning nursing care, there is often a concern for the immediate treatment to surgery and the prevention of complications. This rationale of care denotes a medicalized model, which focuses attention on the healing of a body that is ill, so that the guidelines are based on caring for the body^12^ ^-^ ^14^. The concern of medicine in improving the techniques to correct dysfunctions related to reproductive organs reflects the scientific rationality, which considers the female body as a machine, that presents defective "pieces", and that these pieces must be corrected^13^ ^,^ ^15^, becoming trivial or natural the removal of organs in the name of preserving health. 

In this logic, surgery usually represents the solution of the problems that the diseased organ was bringing to the woman's life, aiming for their physical well-being brought by the disappearance of symptoms^3^. Thus, most of the times the value assigned to this body is not considered relevant, or the beliefs and meanings constructed by it, that should be considered, in order to maintain the mental and social well-being, beyond the physical. 

The psychological association between the reproductive ability, sexuality and femininity started to be studied in recent decades, in order to verify the influence of women undergoing these surgeries in the following aspects: self-concept, self-esteem, sexual and marital relationships, psychosomatic disorders, among others. Several authors^3^ ^-^ ^10^ have discussed these issues, which resulted in findings relevant to the implementation of a comprehensive nursing care to women. 

The loss of an organ related to femininity, whether it is visible or not for others, refers to profound changes in the perception of self as part of the biological body have direct influence on the social structure of the individual, in addition to its own functionality^7^ ^,^ ^16^. There is a need to study the mutilation phenomenon as losing an important part of self and not the pure and simple loss of an organ, not merely in its physical sense. It is a loss that is reflected in the self-esteem, self-image, self-concept and elements that bring the feeling of being a woman. 

This study is based on the fundamental principle of Symbolic Interactionism, according to which human beings act towards things according to the meaning or sense that these things have for them^17^ ^-^ ^18^. Thus, we assume that women act in relation to themselves and to the lived experience of gynecological surgery as the meaning assigned by them to the changes perceived in their body, resulting from these surgeries. Based on this knowledge, this is how they will interact with themselves and with their surroundings.

Therefore, this study had as its object the experience of gynecological surgery by women coming from the loss of reproduction-related organ perspective. The objectives were to describe the sensations and perceptions resulting from gynecologic surgery by women and analyze how they experience the changes caused by the surgery. 

It should be noted that searching in the databases with the words "Mutilation" and "Gynecological Surgeries" points mainly to the result Female Genital Mutilation, indicating that the study of harm associated with the subjectivity tied to the female organs of the body needs to be further explored.

## Method

This is a qualitative, descriptive and exploratory study. It was proposed to unravel processes of interpretation, definition and action based on meanings attributed to a specific situation (gynecologic surgery), by individuals (women) in a context of interaction with themselves and society. 

The Grounded Theory was applied, which is a approach coherent to the theoretical framework^17^ ^-^ ^18^ chosen to support the analysis of the data to be collected ^19^. The research was conducted in a university hospital in the city of Rio de Janeiro, after submission to the Hospital's Ethics and Research Committee (protocol number 2509/2009). 

The inclusion criteria were women undergoing any type of gynecological surgery, and after more than a year since the surgery, because it is a time that reflects not only an adaptation to a new life situation, but a period of repercussions and deeper reflections that could be perceived. Women undergoing gynecologic surgeries as a result of any pathology were included, as long as, regardless of the type of surgery, one or more organs related to the feminine nature or reproduction was removed, in whole or in part, and this was the fact that influenced the experiences studied here. Initially phone contacts and scheduled times were established with the women for interviews from searches in the medical records (searching for benign pathologies) and in the physical therapy schedules of the cancer control clinic.

These criteria were related to the composition of the "sample groups", an expression used by the Grounded Theory, which states that new participants should be recruited to provide the new information that is wished to obtain, due to the inclusion of terms that arose in the analysis^19^. The amount of women included in the first group was determined by spontaneous demand of the first contacts. For other sample groups the criteria were determined by the constant comparative analysis of the collected data^19^. This procedure took place continuously, until it would lead to repetition of the information, constituting the theoretical saturation. There were no imposed limits on the number of participants; data collection was carried out until there was no emergence of new information. 

The interviewees received explanations about the research objectives, the voluntary nature of participation, the confidentiality of the study and the need for recording interviews, they were provided with the Free and Informed Consent Form before the start of the interviews, which took place between March and September 2010.

As part of the study 13 women submitted to surgery: Total Abdominal Hysterectomy, Total Abdominal Hysterectomy with bilateral Adnexectomy, Wertheim-Meigs surgery, Oophorectomy, Salpingectomy, Mastectomy, Quadrantectomy and Tracheloplasty.

The ages of the study participants ranged from 33 to 76 years old, with an average of 47 years. 8 were married, 4 divorced and one was single. Of the 13 women 3 had no children at the interview date. Three women had elementary school, 5 high school education and 5 college degree.

Initially the unstructured interview was applied and from the second group of sample the interview was adapted to direct the collection of new data^19^, then passing the semi-structured interview. This has become more relevant to the continuance of the steps, because the researcher uses a topical guide written to ensure that all issues of research interest are addressed. A guiding question was first proposed to the participants, and other issues were introduced, when necessary, during the testimony. Some issues were introduced after the completion of the first sample group and incorporated into the basic script of the interview, following the method^19^.

As a trigger question of the interviews "Tell me how it was for you to go through surgery" was used. Initially two groups of samples were formed, taking into consideration the type of pathology indicated surgery. In these groups the women were gathered as *women who have had cancer treatment* and *women who treated benign diseases*. After this comparative process two other sample groups were constituted in which women were gathered and *women who withdrew visible organs* (breast) and *women who withdrew not visible organs* (uterus, ovaries and fallopian tubes). In this step we sought to determine whether the loss of an organ or a part of you, visible or not in the eyes of others, influenced the sense of mutilation, regardless of the type of pathology.

## Results and Discussion

Two categories were defined: *Perceiving a different body and feeling as a different person* and *building the meaning of mutilation*

The first category, *Perceiving a different body and feeling as a different person*, It shows that the removal of a body part means to amputate lose something very important for the characterization of self. Some nuances stand out. *She misses more than a piece*, either because she sees the absence on her body or because she has knowledge that an internal organ is no longer there. To explain the surgery performed, she says objectively that an organ was removed totally or partially, and this is a part, a piece of her body. However, this explanation also arose with another intensity, when the woman expresses the strangeness of having a changed body, so that speech is loaded by the *sense of frustration on alien body*, which is now an incomplete body to her. There is also the perception of having a different body as she reports that she has a marked body, and that this mark is a strong symbol of this body change because the scar is interpreted as evidence that shows the difference in her body.

*[...] darn it, I didn't even have a kid and now I have a C-section scar... that's weird. [...] I don't go out there showing my scar to everyone, right? What you can put make-up on, transform, people won't know. It's one less explanation you have to give, you bother less with it.* (E2 - Ooforectomy)

*[...] But it is frustrating, right? Looking at it, and it's not pretty at all! It's a very ugly surgery. I think... It's horrible!* (E8 - Mastectomy)

This different body perception is the concrete dimension of the change that took place, which happened physically. The visible mark on the body in association with the aspects of psychological and cultural nature of the female body refer to traumatic experiences for women^6^ ^-^ ^8^. For this reason the woman expressed a missing piece, she has an alien body or she has a marked body. She sees in herself the body change. Her body, with the features she has, make her as a certain person, interacting in a certain way with the environment.

*Oh yeah, it is a piece of us, right? We are born with all our organs, right? It is a piece...* (E1 - Histerectomy)

*When you look yourself in the mirror, you have one side and you do not have the other. [...] Something so weird... I was shocked.* (E3 - Mastectomy)

*I went home and when I really looked at it, I was sad, but...* (E8 - Mastectomia)

As they go through the experience of the body change, different feelings are experienced, other situations start to make sense and new meanings are constructed. A major change takes place, so that is why the woman is expressing to be a different person. 

*I try to feel normal. Sometimes it's not, but... What else can I do?* (E9 - Wertheim Meigs)

*Felling like a different person*, points out that the change in the body went further, deeper, a fact also pointed out in previous studies^7^ ^,^ ^10^. This duality is well marked in the speeches, which sometimes mingle, and sometimes gets individualized, due to the difficulty, at times, for the woman to measure the influence that a body change has on her.

The *self*, self-awareness is a central concept for the Symbolic Interaction. The self is a social object because when the individual interacts with the conscience, performs the self-interaction and social interaction to define his/her behavior. As the individual interacts with others, the individual defines the self and modifies it continually, society being the context in which he/she develops. The self represents a social process within the individual^17^.

So when the woman expressed that she felt like a different person, this is the result of the interaction process that she experienced, the self-interaction and interaction with others, which enables the *self* to be continuously modified. This self-consciousness changes throughout life, and in the case of these women it changed when they came into contact with a new body reality.

*I mean, I'm not the same I was. [...] Isn't it better to have a healthy person than a deconstructed person? Those who saw me then, and see me now... There is a great difference. People on the streets don't recognize me sometimes...* (E3 - Mastectomy)

This study shows different types of losses: a concrete, physical and other abstract associated with personality and identity. Hence it is observed that the woman relates her loss with subjective spheres, related to the construction of the image of herself.

*I did it, I have one ovary less.* (E2 - Ooforectomy)

*So, that's it... you swallow like water, you know how it is? That's a hard pill to swallow? [...] These things are not easy for me [tears]. I try to work it out in my head. [...] It's not something I settled yet.* (E4 - Salpingectomy)

The self-image or ego identity is a psychological need that refers to how a person perceives herself consciously, that is, what she sees and thinks of herself. It is also a dynamic and multifactorial construction, which requires frequent restructuring and affects the way you relate socially^4^ ^,^ ^6^ ^,^ ^8^. 

*[...] because I didn't showed it, I had a lot of... It wasn't shame, I don't even know what it was, but I didn't show. I spent a whole year not showing it [...] because I had a body, and now I have another... And by less, right? In my head at least.* (E5 - Mastectomy + Histerectomy with Adnexectomy)

The interviewees notice themselves as different people specifically by modifying the image they have of themselves after surgery. This was gradually taking place, from the observation of others' attitudes, comparing their new body with other women and assessing whether they can still perform the same functions as before. This is nothing more than part of the process of interaction that determines the course of action of each person^18^. So, they noticed that the change in the body interfered with psychological and sociocultural aspects, which required new stances - from a new woman.

A negative connotation of feeling like a different person marked the speeches, when women attributed the meaning of devaluation of themselves associated with the changes in their body. Such circumstances lead to the rejection of themselves, and it could lead women to feel inferior, excluded or disfigured^6^ ^,^ ^8^ ^,^ ^20^. They referred feeling deconstructed, abnormal, incomplete, which leads to the importance certain "body parts" - uterus, ovaries, fallopian tubes, breasts - have for the subjectivity of being a woman and feeling like a woman as a whole.

The value the removed organ has to the woman is historically rooted in social and cultural constructions, which outlined certain characteristics as natural and inherent to women based on biological characteristics. As society is composed of interaction groups, it is understood that this process determined that, over time, roles and obligations are inherently associated to each sex. Thus, the female nature is mainly based on biological facts that occur in the body of the woman: ability to engender, give birth and breastfeed, as well as menstruation^13^ ^-^ ^14^, facts socially and culturally understood as fundamental characteristics of women. A fundamental feature for the construction of which is to be a woman, the loss of reproductive organs and the ones involved on motherhood are able to deconstruct this historical feminine image. 

This idea is complemented by making a full association of motherhood with the constitution of femininity and identity of the woman, based on socio-cultural parameters described above. As the feminine nature is essentially maternal and reproductive, her own female sexuality is related to these qualifications^9^ ^,^ ^14^.

All of this enables the understanding of what being a woman is, a derivative symbol of the process of social interaction^17^, and explains why this strong sensation of being *different people* emerging from the interviewed . They did not define that they are no longer women, but they compare themselves to other women and feel undervalued, which means that they feel like *different women.*

*I think that there is an appreciation from society related to aesthetics. For example, I see women in INCA that do not wear prosthetics.. They go out on the street without their prosthetics. And it is clear that people look at them and get shocked. [...] You become "different" if you do not use an external prosthesis.* (E5 - Mastectomy + Histerectomy with Adnexectomy)

The devaluation felt by women were related to the function that that body has lost and that they no longer possess. If the body had a purpose and a role that marked this female identity, after the surgery the function that women attributed to themselves was changed or diminished, belittled. Each organ was explicitly given a functional reference by the interviewees: (1) the ovary produces hormones and enables the female fertility; (2) the tube is conductor of the egg, also allowing fertility; (3) the uterus provides the ability to engender and develop the babies as well as being associated with the ability to menstruate; and finally (4) the breast is related to breastfeeding, femininity, sensuality and motherhood.

So, while I was at the time of the process, you do exams, do this, do that, I did not feel it so much. But after you do it, then what? [...] You already start to think ... the difficulty to get pregnant will increase, it is an ovary less. [...] Today I still worry because I don't have a son yet [laughs]. (E2 - oophorectomy)

I think a finger, one little finger you lose is important. Because there are people who won't accept it, that keeps looking at you. (E3 - Mastectomy) 

Of course it is not good to be without a part of our body. It's bad. But what is there to do? It's good to have everything that is mine. (E9 - Wertheim-Meigs)

The associations made by women who went through gynecological surgery - to perceive the changed body, of feeling like a different person, to have functional, social and sexual interferences - contributed to the construction of a special meaning to the mutilation felt by participants, the second category *Building the meaning of mutilation.*

The mutilation was divided into two dimensions: a concrete one, given the loss of the organ, and an abstract one, formulated from the impact that this event had on various aspects of their lives. This phenomenon, assuming the feature of amputation, deprives women of an organ, which is marked on their body; the mark is the physical proof that the loss occurred^1^ ^,^ ^15^ ^,^ ^21^. Another sense of mutilation is defined as a mutilation of the feminine personal identity, as the identity they begin to see in themselves is modified due to the loss of an organ that is characteristic of every woman^3^ ^-^ ^7^ ^,^ ^9^. The reflection of mutilation made by participants points to the perception of physical, social and functional mutilation felt by them. The female identity has its origin in the feminine characteristics (biological), which in turn built a social identity for women, based on the functions performed by them^14^.That is to say, when a woman feels that her social and functional identity has changed, it is because she has built a new outline for their own way of perceiving herself and build their feminine personal identity.

*Being an organ that has a function, its removal is a mutilation. [...] There is a limitation. As much as people try to convince me otherwise, and I try to work it to convince me too, I see it like this, you know? [Voice breaking] Because if it did not have a function, you do not need it, like an appendix. (E4 - Salpingectomy)*

Thus, it can be said that the socially mutilated woman is one that had her social body mutilated, by perceiving this body as a tool to relate to the world^8^, a body which is also cultural and historically constructed. The functional mutilation applies to the loss of the function designated by the removed organ, which in turn interacts with your way of relating socially, demonstrating that both feelings are mixed.

Given the subjectivity of perception by individuals^9^ ^,^ ^16^, the interviewees of this study demonstrated that the mutilation was relative and changeable. It was related to *the feeling of realizing a piece is missing*, and this also depended on the body's location. Both internal or external, several women "saw themselves" missing a piece, as part of the image of themselves is physically built and the other is abstract. Thinking about the composition of the body is enough to understand if she has a part of it or not^8^ ^,^ ^16^, and the mutilation is guided by this understanding and the impact that this phenomenon brought to the woman.

*It is [hysterectomy is a mutilation]. To a lesser extent, but it is. I think it's due to the issue of valorization. It has no comparison. The breast is a mega mutilation, the uterus is a minimum mutilation. Got it? (E5 - Mastectomy + Hysterectomy with adnexectomy)*

*It is a part that is out, that was removed, you see? So I think it is mutilation because of this. Taking any part of the body is a mutilation. (E8 - Hysterectomy + adnexectomy)*

There is a trend in academic research on calling mutilation a mark that can be seen with the eyes, an act performed in the body that aims to eliminate the disease and which is necessary to heal the individual. As an example, the mastectomy is widely characterized as mutilation different from other gynecologic surgeries. This broadening of understanding is still achieved timidly in other studies on hysterectomy, in which some authors have used the mutilation expression to explain aspects of surgery^3^ ^,^ ^21^ ^-^ ^22^.

To relate to other people who perceive their new body as defective, the woman often comes to believe that it has an imperfect body, experiencing feelings of inferiority^6^ ^,^ ^8^ ^,^ ^16^. The integration of women to their social environment, supported by stimulus, encouragement and help, held by family, friends or even a support group is a strategy to overcome the obstacles and limitations faced by the sickness and by living with mutilation are minimized^23^ ^-^ ^24^.

By representing the woman's body after surgery as defective, their social environment assigns a symbol, which explains how what was seen was interpreted. This interpretation of the group was mediated by influences of interactive source with others and past experiences. The woman, realizing her body as a target of this symbolism (defective, mutilated, incomplete), absorbs these opinions and interacts with this information. The self and the mind conceive their own meaning for the woman, which is the result of the interaction that occurred with herself and with her social environment, and from that it defines other situations for herself and elaborate her actions^18^. 

*It's very different, because of the valorization. My valorization! So then, the breast is one thing that I miss. [...] for myself. (E5 - Mastectomy + Hysterectomy with adnexectomy)*

The fragmented care model currently offered to the population^3^ ^,^ ^22^ ^)^ has shown that does not meet the needs of a woman who undergoes a gynecological surgery, it does not treat her as a whole woman, a woman who associates the surgical procedure with the social valorization of her body, femininity, sexuality, mutilation and other aspects. 

This way of fragmenting the body, overvaluing the biological component also leads to fragmentation of care^13^ ^,^ ^15^, so that the nursing profession itself often turns out to be limited in providing technical assistance - to the biological body - without being able to provide the real care, that reaches out to the remaining human dimensions.

Due to this difficulty in understanding that the female reproductive organs are part of a broader feminine individual perception, exploring the problem of experiencing changes after gynecological surgery is a challenging matter. The most recent publications on the subject are scarce, indicating that often the reality here mentioned by women is not even seen or reflected by professionals as a possibility of acting or giving care to women. And the interviewed, despite having spent more than a year after the completion of surgery still wanted to talk about their experiences, and also presented care demands.

In this sense, the results of this study call the attention of professionals to the biological, physical and social aspects of the illness, which reflect the comprehensiveness of care to be provided. They also brought important data for the research field related to women's health care, and point to the need to develop further research in this area. It is also necessary to reformulate the way of teaching professionals to take care for the other person, beginning with the non-fragmentation of the human being.

## Conclusion

As for the feelings and perceptions arising of gynecologic surgery, this study showed that after some time of surgery the women had reflected on what surgery meant and how their lives would be from then on. We observed an association of physical loss with the construction of the image of them. Regarding the way to experience the changes caused by the surgery, when realizing there was a different body: missing a piece, a estranged body and a marked body, women interacted with themselves and their social environment and resented the loss, modifying the image they had of themselves and perceived themselves as a different person. Thus this came to reassess their value as a woman and reflected on the mutilation: physical, social and functional. The changes experienced are the result of a process of interaction with different social objects, which enabled new directions to be formulated.

This understanding points to the importance of a care that integrates the various dimensions of the human being, seeing woman as a whole. This kind of care respects the fact that there is a psychological and social context when the disease is treated. When addressing the woman receiving care in the context of gynecologic surgery, the nurse should be able to understand that individual and sociocultural factors interact to form the understanding of women on the process experienced with surgery. Knowing these meanings for her and the way she interprets what happened to her is an important tool for care. From this perspective, the nurse can be a key agent to help women find their own way to overcome the difficulties they face.
